# Systems Pharmacology in Small Molecular Drug Discovery

**DOI:** 10.3390/ijms17020246

**Published:** 2016-02-18

**Authors:** Wei Zhou, Yonghua Wang, Aiping Lu, Ge Zhang

**Affiliations:** 1 Institute for Advancing Translational Medicine in Bone & Joint Diseases, School of Chinese Medicine, Hong Kong Baptist University, Hong Kong, China; z_wei1005@126.com; 2 Institute of Integrated Bioinformedicine & Translational Science, School of Chinese Medicine, Hong Kong Baptist University, Hong Kong, China; 3 Department of Scientific Research Management, Shanghai Guanghua Hospital of Integrated Traditional Chinese and Western Medicine, Shanghai 200052, China; 4 Institute of Basic Research in Clinical Medicine, China Academy of Chinese Medical Sciences, Beijing 100700, China; 5 Center of Bioinformatics, Northwest A&F University, Yangling 712100, Shanxi, China; yh_wang@nwsuaf.edu.cn

**Keywords:** drug discovery, systems pharmacology, ADME/T, network pharmacology

## Abstract

Drug discovery is a risky, costly and time-consuming process depending on multidisciplinary methods to create safe and effective medicines. Although considerable progress has been made by high-throughput screening methods in drug design, the cost of developing contemporary approved drugs did not match that in the past decade. The major reason is the late-stage clinical failures in Phases II and III because of the complicated interactions between drug-specific, human body and environmental aspects affecting the safety and efficacy of a drug. There is a growing hope that systems-level consideration may provide a new perspective to overcome such current difficulties of drug discovery and development. The systems pharmacology method emerged as a holistic approach and has attracted more and more attention recently. The applications of systems pharmacology not only provide the pharmacodynamic evaluation and target identification of drug molecules, but also give a systems-level of understanding the interaction mechanism between drugs and complex disease. Therefore, the present review is an attempt to introduce how holistic systems pharmacology that integrated *in silico* ADME/T (*i.e.*, absorption, distribution, metabolism, excretion and toxicity), target fishing and network pharmacology facilitates the discovery of small molecular drugs at the system level.

## 1. Introduction

Drug discovery is a long and extremely complicated process with a low success rate and vast capital investment. Recent advances in chemistry have promoted the efficiency of chemical compound synthesis, by which the chemical libraries can generate and store total amount and whole diversity of data. Although the high-throughput screening of various molecules has processed considerable development to identify major compounds with activity therapeutically against targets and pathways, the number of successfully identified molecular drugs did not significantly increase over the years [1]. The evidence shows that the human body is a complicated and integrated system that is composed of various scales of biological organization, from molecular to organismal, which will make the full rational drug design process and systems-level understanding of drug action extremely difficult. Molecules that are hopeful in cell-based assays commonly do not work or present unfavorable pharmacokinetic properties or cause adverse effect *in vivo*. Moreover, most new drugs frequently fail in Phase II and Phase III clinical trials because of the complex relationships between drug-specific, human body and environmental factors influencing drug response. Therefore, there is a pressing need to develop an innovative and integrated system-level approach to systematically and comprehensively parse the mechanism of drug action so as to deep understand the interplay between drugs and complex disease.

In recent years, systems pharmacology has drawn an increasing focus as a multi-interdisciplinary subject, including drug pharmacology, systems biology, physiology, mathematics and biochemistry. Systems pharmacology is a global approach to translational medicine with the purpose of clarifying, validating and using new pharmacological concepts to the development and application of small molecule and biologics [2]. The significant potential of systems pharmacology is that it offers an integrated system-level method to identify existing and new drugs interindividual drug variability, and it facilitates prediction of effectiveness and security of compounds during all phases of drug development. There is a growing appreciation that the well-recognized definition of a successful drug is properly balancing potency, efficacy, safety and favorable pharmacokinetics. During the process of drug discovery, one challenging issue is the the knowledge required to develop drugs is always inadequate due to the complexity of drug–body interaction as well as the complexity of individual response to drug perturbation. To avoid late-stage failures in the discovery of new chemicals to be used as drugs, ADME/T (*i.e.*, absorption, distribution, metabolism, excretion and toxicity) studies including absorption, distribution, metabolism, excretion, and toxicity are involved in a much earlier stage of the discovery process [3,4]. Moreover, drugs play imperative roles in the therapy of a disease and drug exploration focuses more on the validation of potential targets which may exert great impact on disease genes [5]. It is also extremely important to dissect the mechanism of drug action by considering targets that exist in the context of biological networks. The analysis of the biological networks associated to a given disease can identify potential drug–target interactions to achieve the desired outcome at the system level.

**Figure 1:**
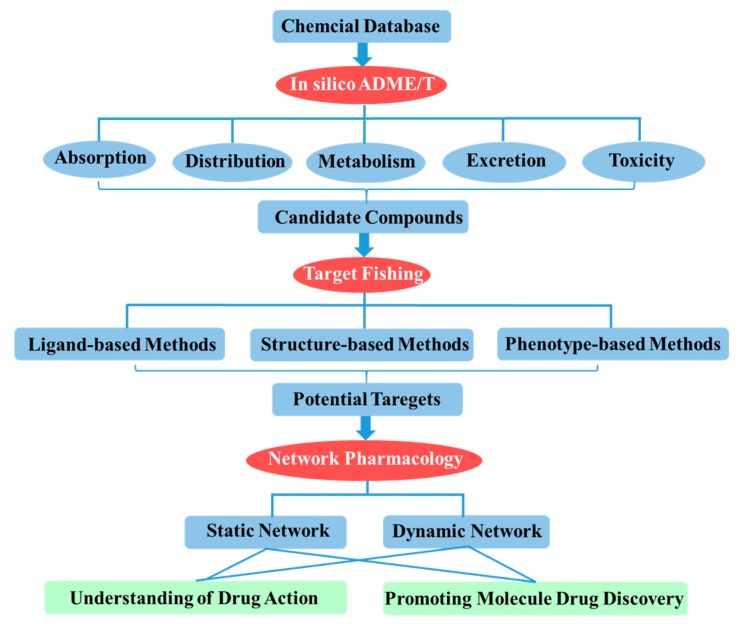
Flowchart for systems pharmacology-based drug discovery.

Therefore, in this review, we provide a holistic systems pharmacology strategy that integrated *in silico* ADME/T, target fishing and network pharmacology for the discovery of small molecular drugs at the system level (Figure 1). This comprehensive approach not only focuses on addressing disease mechanism, identification protein target and approaching drug discovery, but also aims at highlighting the invaluable role that system-based methods have played, and continue to play, in the drug discovery process and its future perspectives.

## 2. *In Silico* ADME/T (Absorption, Distribution, Metabolism, Excretion and Toxicity) Assessment for Drug Discovery

The development of combinatorial chemistry and high throughput medicinal chemistry programs give us more opportunities to synthesize a large amount of compounds at much shorter term than conventional medicinal chemistry. However, it has been reported that 95% of drug candidate molecules fail in the development stages, and 50% of such failures are caused by unsatisfactory ADME/T properties [6]. ADME/T properties have been considered to be a major reason for the failure of candidate molecules in the late drug discovery stage, and this has led to increasing interest in identifying such problems earlier in the drug discovery process. To avoid such failure, a set of *in vitro* ADME/T screens have been implemented in many pharmaceutical companies with the aim of identifying and removing compounds with poor ADME/T profiles as early as possible in the process of drug discovery. Although advances *in vitro* ADME/T techniques have decreased the probability of the failure at the drug development stage, it is still time-consuming and resource intensive. Thus, it is necessary to develop *in silico* methods that are faster, simpler and more cost-effective for evaluating the ADME/T properties of a one single molecule in advance. The *in silico* prediction of ADME/T characteristics is an attractive alternative to experimental measurements because it provides an easy accessible high throughput method to improve the ability of screening and testing by only focusing on the promising compounds so as to reduce time and expense of the drug discovery process. The exhaustive list of ADME/T models is described below.

### 2.1. Absorption

Absorption is the transfer of a drug from its site of administration device directly into the bloodstream and is not required when a drug is administered intravenously. Drug absorption is a complex process that is dependent upon the route of administration, the formulation and chemical properties of the drug, and physiologic factors that can impact the site of absorption. Oral medication is the most ideal route of drug administration, hence, there is great interest in the prediction of intestinal absorption and intestinal permeability. 

*In silico* models of drug absorption can be categorized into two categories, *i.e.*, physicochemical and physiological. Some simple filter approaches used to the evaluation of some physicochemical properties have been proposed, including aqueous solubility (logS), logarithm of octanol-water partition coefficient (logP), logarithm of octanol-water distribution coefficient (logD), acid dissociation constant (pKa), *etc.* that have been widely used and easily identified bioavailable drugs [7,8]. However, the evaluation of the intestinal drug permeability cannot be accurate solely based on physicochemical elements because of the presence of multiple drug transport pathways. For the screening of many molecules, the physiological models are needed. Human Intestinal Absorption (HIA) as key procedure of oral absorption is one of the most influential ADME/T properties that evaluate the success/failure of a drug candidate during development process. Many computational classification and correlation models have been developed to predict the HIA based on a large amount of data that are produced rapidly by *in vivo* and *in vitro* experimental assays [9,10]. In addition, among ADME/T properties, good oral bioavailability is often one of the most desirable attributes of a new drug, which refers to the rate and extent of absorption. For instance, based on 805 structurally diverse drug and drug-like molecules, a robust *in silico* model was developed to predict human oral bioavailability based on self-organizing maps [11].

### 2.2. Distribution

Distribution is defined as drug diffusion or drug transfer from intravascular space to body tissues. Once the entrance of a drug molecule to the systemic circulation takes place, the drug is distributed by the blood flow to different organ systems and tissues all over the body. The distribution of a drug molecule throughout the body is a pivotal determinant to understand because it is usually a prerequisite for the movement of a drug molecule from the blood into other tissues before it can drive its pharmacodynamics or toxic effect. Thus, the prediction of drug distribution in drug development would benefit the pharmacodynamic and toxicodynamic effects assessment in certain tissues before any experiments in animals or man. Currently, several methods are available to predict drug distribution, which involved prediction of plasma protein binding (PPB) or blood–brain barrier permeation (BBB) [12,13]. PPB is an important parameter to the distribution modeling, because it may lead to less bioavailability and inadequate drug–drug interactions [14]. A representative *in silico* model to predict PPB is the web application of Zsila *et al.* [15] based on SVM-aided docking approach, which showed a good predictive power.

A series of specialized barriers have been identified and characterized in different parts of the body that protect the vital organs and systems of the organism against hazardous chemicals that can enter the body intentionally or unintentionally. The BBB is one of the most important barriers specialized to protect the central nervous system (CNS) and is located at the brain capillary endothelial lining. The BBB plays a crucial role in isolating the brain from the bloodstream, and the vital limitation for CNS drug discovery is the challenge in designing molecules able to cross it. The distribution of potential drug molecules cross from the blood into the brain depends on the ability of molecules to penetrate the BBB. A good example that exhibits the great practicability of a predictive computational model is the BBB permeability model, which can help to facilitate early screen of molecules with low BBB penetration profile in advance, therefore will have a profound effect on drug discovery and development. Some *in silico* models have been developed to measure the potential for novel molecules to permeate the BBB based on the assumption that molecules are transported across the BBB by passive diffusion. By far, the ratio of drug brain concentration/drug blood concentration (LogBB, BB represents brain-blood) is the most used parameter for predicating BBB penetration and the higher ratio shows higher concentration in the brain [16]. Recently, Carpenter *et al.* [13] developed a simple BBB mimic based on MD and binding free energy approaches for logBB prediction.

### 2.3. Metabolism

Among the ADME/T properties, metabolism is probably the most challenging one to evaluate and predict, considering the complication of related multiple enzyme systems. Metabolism is an important process in determining the formation of metabolites of a drug in the body, which has implications for its safety and efficacy. Particularly, metabolism can play a key role in a number of issues, such as poor bioavailability because of enhanced clearance; toxic effects produced by drug accumulation; and drug–drug interactions, including enzyme inhibition, induction, and mechanism-based inactivation [17,18]. Orally absorbed drug is transported through portal circulation to the liver, which contains the necessary enzymes for metabolism of drugs and other xenobiotics. The drug metabolism is very complicated because it involves several metabolizing enzymes which can be classified into phases I and II. The cytochrome P450s (CYPs) as Phase I enzymes are contributed to ~90% of the metabolism of drug molecules, whereas metabolism and several other enzymes, such as UDP-glucuronosyltransferases, sulfotransferases and methyltransferases at the Phase II metabolism [19].

Recently, a variety of *in silico* modeling techniques have been used for the prediction of metabolic products based on the approaches including expert systems, quantitative structure-activity relationships (QSARs), molecular interaction fields (MIFs), and protein-ligand docking [18]. Primarily, expert systems are built and maintained through codifying the metabolic reaction rules from the literature, books, and patents, which have been widely employed for the prediction of drug metabolism, mainly contain some commercially available like METEOR (is an expert method to anticipate the possible metabolic fate of compound based on its chemical structure) [20], MetabolExpert (is an expert system for predicting metabolism of substances) [21] and META (is a knowledge-based expert system stimulating the biotransformation of xenobiotics) [22]. All knowledge-based categories of biotransformation reactions in both phase I and phase II are involved in these systems, meanwhile include a series of rules reasoned by variety levels, executing the knowledge of experts systems in the process of drug metabolism.

In general, most of the studies have been developed to predict phase I metabolism reactions involving CYPs. The three dimensional structure of CYPs from X-ray crystallography has facilitated the molecules docked into CYPs active site to figure out the available binding modes and metabolic sites. Moreover, as one of the most attractive methods in evaluating histone based metabolism, the structure-based approach has been successfully applied on a number of macromolecules related to the ADME/T processes [23].

### 2.4. Excretion

Drug excretion is the process whereby a drug molecule is eliminated by liver, kidney and other organs from the body. Commonly, the excretion of drug molecules occurs through two main routes: via urinary excretion in the kidneys, and via biliary excretion in the liver. The excretion pathways directly influence the amount of available drug molecules to interact with the biological target, as well as the half-life time and the administered dose.

Although passive excretion can theoretically be predicted based on a great many physicochemical and physiological properties, such as the blood flow, protein binding and lipophilicity [24]., those that are restricted to a significant extent by these properties may have different limits, such as glomerular filtration and molecular weight. Renal excretion is a composite of a number of different processes including glomerular filtration, active secretion and reabsorption each of which driven by different properties. The complexity of renal excretion has hindered the development of *in silico* modeling or prediction of excretion to date. Recently, based on 349 drug molecules, Paine *et al.* [25] applied Partial Least Squares and Random Forest to predict the human renal clearance and the Random Forest model showed superiority in all statistical parameters (fitness, robustness and predictability). In addition, Hsiao *et al.* [26] predict clearance of 244 drug molecules by comparing the Random Forest with other methodologies (Orthogonal Partial Least Squares and Multiple Linear Regression). The result showed that Random Forest model also exhibited better results than the other two methods.

### 2.5. Toxicity

Arguably, drug toxicity is the most challenging drug property that remains one of the most significant reasons for many drugs failing to reach the market and for many drugs not approved to the market and withdrawal from the market during the late-stage drug development. The drug toxicity is a complex biological process because it can occur at organelle, cellular and systemic levels and can result from receptor/enzyme or DNA interaction, induction or inhibition of hepatic metabolism, as well as the combination of several causes, all of which cannot be satisfactorily modeled experimentally. A critical priority in drug development is the early period identification of pestilent toxicity in case of wasting time and resources in late stage.

The development of toxicity testing’s alternative approaches attracts more interest due to their relatively simplified implementation and flexibility, providing considerable benefits such as high throughput, low expenditure and relatively less time of analysis [4]. Need for the improvement of *in silico* virtual models and for a more detailed knowledge of the effects of various chemicals on physiological mechanisms. This strategy could then be applied for toxicological assessments during earlier stage of the drug discovery process and for evaluation of more candidate drug molecules [27]. Recently, some integrated methods providing all-sided prediction in the early stage and coupled with decision-making have emerged and enhanced the production ratio of subsequent drug development steps. The existing software packages including DEREK (an in slico expert system for the qualitative evaluation of likely toxic action of compounds according to their described chemical structure), Hazard Expert (is an essential software for quick toxicity estimation of organic compounds), OncoLogic (is an ideal program to predict chemicals that may cause cancer), TOPKAT (Toxicity Prediction by Komputer Assisted Technology), MCASE (a multiple computer automated structure evaluation) and PASS (Prediction of Activity Spectra for Substances) are commercially accessible for the prediction of potential toxicity [28,29,30]. For instance, as the first earliest toxicity prediction software, DEREK uses a classic knowledge-based expert system from human experts and the scientific literature [31]. And MCASE relies primarily on a machine-learning method to recognize molecular fragments with a high possibility of being connected with observed activity [32]. Moreover, some *in silico* models for the predicting of preclinical drug toxicity studies have been developed. Based on 288 drugs or drug-like compounds, Zhang *et al.* [33] successfully constructed a classification model to predict mitochondrial toxicity by using Support Vector Machines. Myshkin *et al.* [34] constructed several classification models to predict the organ toxicity of compounds using Decision Trees method and showed good performance. Several properties including general hepatotoxicity and nephrotoxicity, as well as specific liver and kidney necrosis, liver and kidney relative weight increase, liver lipid accumulation and nephron injury were evaluated by these models. Despite *in silico* toxicity prediction or simulations are ponderable for drug discovery and development, more effort should be paid on the improvement of their prediction accuracy and mechanism interpretability.

## 3. Target Fishing

Drug discovery for complex diseases focuses more on recognition of the drug targets that can be utilized to produce the therapeutic effect while not allowing unwanted side-effects aroused by off targets. A major property required of an ideal drug target is that the biological rationale of its use must be obvious. An ideal drug target modulated by a small molecule could be defined as a macromolecule (most often a protein) whose manipulation could result in removing the causes or relieving the symptoms caused by the underlying pathophysiology. During the past, drug design conception was restricted by the so-called “one drug-one target” approach because of the complexity of biological systems. [35]. This concept states that most selective drug molecules exert their activities by acting on individual targets particularly related to a disease, famous for the analogous saying as one “key” (or ligand) modifying each “lock” (or protein) [36]. However, in recent years, this paradigm neglects the cellular and physiological circumstances of the drugs’ mechanism of action existing which fails to develop better drugs with satisfactory therapeutic effects expected to treat various diseases [37,38,39]. Increasing evidence that many drugs exert their therapeutic activities by modulating multiple targets is accelerating the development of research fields in objection to the data reductionism approach [40]. It has become obviously suggested that the existence of a better paradigm, the central idea of which is rooted in the multiple drug-multiple target principle [41]. In spite of being advanced for target identification, the wet lab experiments are still insufficient in terms of expenditure and effort (various activity assays for each protein, difficulties in protein isolation, *etc.*). Considering the tremendous growth of bioactivity databases, the use of computational methods to predict drug targets of small molecules has become increasingly important in recent years. Presently, the most widely used computational methods for drug target identification can be approximately classified into three groups: ligand-based virtual screening, structured-based virtual screening and phenotype-based (Table 1) [42].

### 3.1. Ligand-Based Methods for Protein Target Prediction

Ligand-based methods are extrapolated from known bioactivity compounds utilized as input information and aim to predict the effect of new compounds based on the properties of compounds known to bind to the desired targets. The hypothesis underlying ligand-based virtual screening assumes that chemical structures similarity between drugs tends to present similar targeting activities, though various compounds similar in structures may interact with targeted protein in various ways [43]. Ligand-based virtual screening can be performed using different simple filters, such as one-dimensional (1D) filters (e.g., molecular weight), two-dimensional (2D) filters (e.g., substructure matching), or three-dimensional (3D) filters (e.g., 3D similarity or pharmacophore filters). A widely-recognized example is the QSAR method (Quantitative Structure Activity Relationship), which uses two-dimensional (2D) topological fingerprints encoding atom types and their bond connectivity. Molecular fingerprints of small molecules can consequently be used as feature vectors to train statistical regression or classification models to predict their binding activity towards specific target proteins [42,44].

Nidhi *et al.* [45] applied Multiple-Category Bayesian model to distinguish chemical compounds based on their targets. A striking development in related proteins on the basis of the distribution of characteristics in each bioactivity set of molecules is the development of the similarity ensemble approach (SEA), which utilizes a BLAST-derived algorithm to exploit minimal spanning trees in the consideration of chemical similarity [46]. SEA estimates target similarity by normalizing the sum of similarity scores between two groups of ligands recognized to bind to their targets and several SEA predictions have been experimentally certified [46,47]. Calculating the similarity of over 3000 FDA-approved drugs coupled with hundreds of targets based on large scale, 23 new interactions between drugs and targets have been uncovered. Based on *in vitro* experiments, five of them were confirmed to be potent with affinities less than 100 nM [48]. In addition, the potential off-target effect of some commonly used drugs targeting protein farnesyltransferase (PFTase) was also investigated by SEA method [49]. Although the small molecules topological representation was succeeded, ligand-based approaches are generally not applicable in cases if no ligands for a specific protein exist, since in these cases no training on ligand-based information is feasible. To be used most effectively, ligand-based methods require enough known ligands for targeting proteins of interest, which may be hardly accessible in practice [41].

### 3.2. Structure-Based Methods for Protein Target Prediction

Structure-based methods generally elucidate approaches that develop protein structural information associated with scoring functions to predict the protein-ligand binding mode, thus providing valuable insights for better drug selectivity enhancement. The central goal of this approach is to assess a molecule’s ability to bind with a specific protein and to exert a desired biologic effect depends on its ability to favorably interact with a specific binding site on that protein. When the 3D structure of the biological target is available, structure-based methods are reliable for providing a molecular framework representative of the essential physiochemical features required for biological activity of the inhibitory compound. 

Several recent studies have combined targets sequence features with fingerprints of ligands to train models based on statistical machine learning for target prediction [50,51]. The most extensively used form of structure-based target prediction methods is protein-ligand docking, which predicts preferred interacting site when drug candidates target potential specific proteins [52]. Structure-based methods involve molecular docking of each ligand into the binding site of the target followed by applying a scoring function to assess the likelihood that the ligand will bound to the protein with high affinity [53,54]. Recently, a study of currently available methods for *in silico* reverse screening used for target prediction was performed by Kellenberger *et al.* [55] with the purpose of examining how the algorithms were performed for determining the level of protein targets perform. Moreover, several ranking approaches based on the comparison of GOLD fitness score and topological molecular interaction fingerprint (IFP) were appraised in that study, since it was found scoring functions still the main weakness of virtual screening approaches. The result showed that problems associated with accuracy and false positives were shown to be addressed. Obviously, these methods are rely on available 3D protein structures which are of limited utility on a genome-wide scale [56]. Despite structure-based methods have made great advances and breakthroughs in target prediction, much more efforts are still required to overcome the limitations for attracting a wider acceptance.

### 3.3. Phenotype-Based Methods for Protein Target Prediction

Phenotype-based methods are a prominent part of the drug development process used for identifying drug candidate compounds with a desired phenotype [57]. However, the molecular mechanisms of the hit compounds remain unknown when it is used alone. Thus considerable effort is required to identify the target proteins associated with the phenotype. For the past few years, a number of methods from wide fields have been explored to identify targets from phenotypic information. In particular, computational approaches have become more powerful to identify multiple proteins and relevant pathways that may not have been previously linked to a given biological output based on phenotype changes [58].

The interactions between drugs and their targets are linked through their phenotypic information [59], although the relevance between similar drug phenotypic responses and similar drug actions is not always great [60]. Phenotype-based methods associate different drugs by analyzing the biological phenotype responses, such as gene expression profiles in cell lines or proteomic data [42]. Several reviews have evaluated the recent advances in using genomics- and proteomics-based methods to establish drug–target relationships [61]. A wide spectrum of computational methods including machine learning, statistical analysis and network analysis can be used. Seminal work is the national NCI-60 project, which selected and analyzed 60 human tumor cell lines coupled with more than 100,000 related compounds to construct a database recording the basal gene expression and drug sensitivity knowledge [62]. Recently, Iskar *et al.* [63] developed a computational normalization and scoring procedure to screen and establish drug response gene expression profiles. The drug-induced gene expression profile can be used not only to establish a global drug-disease network for the investigation of drug mechanisms [64], but also to identify common disease modules and pluripotent targets [65]. It is reported that other phenotype information including cell imaging have also been utilized to connect different drugs and to speculate about their potential targets [66,67]. For instance, based on the image-based cellular phenotypic screening, Young *et al.* [67] developed a factor analysis method to profile chemical compounds. Using text mining approach, Campillos *et al.* [66] utilized a molecular signature associating English terms with drug side effects to connect a drug with its unknown targets. It is to be expected that more and more phenotype information that can be applied in basic biology and drug discovery research such as drug–target and pathway-disease interactions will be generated from phenotype screening experiments in the future.

## 4. Network-Based Drug Discovery

In conventional drug discovery, the philosophy of drug design has relied heavily on the single-drug-single-target paradigm. However, in real biological systems, it has been appreciated that many effective drugs act on multiple targets, which can form complex interaction networks included in cellular state regulation. The complication of biological systems has limited this paradigm of drug design since there are multiple interactions between the genes and other molecules instead of the change of a single gene. It is thus extremely difficult to discover better drugs with therapeutic effects expected to treat various diseases because of the enormous complexity of various networks involved in various disease states [68,69]. Therefore, network pharmacology-based approaches that consider such biological complexity are the driving force behind a new concept in drug discovery.

Regarded as the technical route to the ultimate ideal of systems pharmacology, the network pharmacology aims to identify a set of drug targets for any disease by network analysis and investigates the effects of the drugs binding to these targets by chemical biology approaches. Network pharmacology has the potential to accelerate drug discovery through the identification of connectivity, redundancy, and pleiotropy in biological networks [70]. A network pharmacology-based approach to the prediction and analysis of the interactions between a drug and its targets not only develops a systems-level comprehension of drug action and disease complexity, but also helps to improve the drug design efficiency. Generally, network pharmacology-based approaches can be divided into two categories: static network and dynamic network.

### 4.1. Static Network

**Figure 2:**
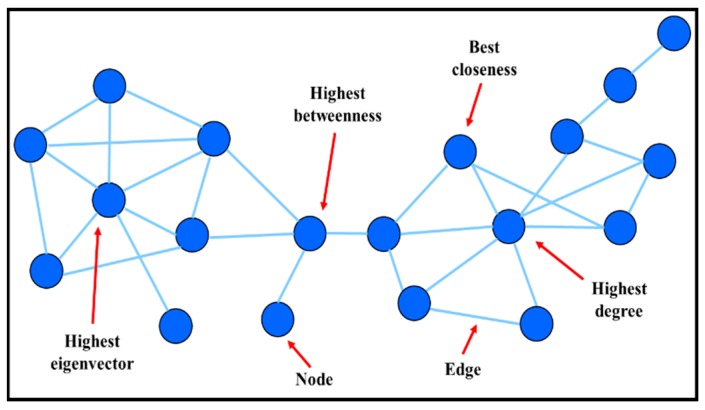
Topological structure of static network.

In a topological sense, there are two main components of the static network include entities (“nodes”) and modeling the relationships (“edges”) (Table 2, Figure 2). The nodes are the vertices in the network that represent different types of objects such as genes, proteins, small molecules, molecular pathways, disease or any other entity with interacting in the modeled system. Edges are the pairwise interactions between the nodes that represent protein–protein interactions, drug–target interactions, target–disease interactions or transcriptional regulation. When the information is accessible, directions, weights and other attributes can be shown in edges; therefore, understanding about hierarchy of effects will be developed. 

Topological features of a network are significant for understanding and accessing the performance, robustness, and scalability of network protocols and applications. Application of several basic topological properties recognized as “centrality” contributes to the local characterization of networks which include degree, betweenness, closeness, and eigenvector centralities (Table 2, Figure 2) [71]. As a fundamental quantity representing the topology of scale-free networks, degree is one of the commonest centrality measures [72]. The degree is the number of edges connected to a node. A molecule interacting with many other distinct molecules would have a high node degree, the so-called “hubs” [73]. In scale free networks, a power-law distribution is followed by the node degree, which conveys us how much access a particular node has to the other nodes. Betweenness is yet another commonly used measure of centrality that describes the influential level in node pairs communication [74]. In other words, betweenness is defined as a measure of the number of shortest paths that pass through each node. It is important for finding non-hub important nodes [75] or classifying hubs according to their positions in the network. The reciprocal of farness is closeness centrality which could assess the time required for information to transmit to a given node in a network through calculating the length of the path between them. This feature is usable only in connected networks because of the distance ambiguity between unconnected nodes. Eigenvector centrality, which is a measure of the impact of a node in a network, is not restricted to the shortest paths. It can be used for evaluating relative scores of all network nodes on the basis of the connections to high-scoring nodes leading more to the problematic node score compared with low-scoring nodes. In conclusion, these centrality measure may help us to get clearer insight about the topological properties of the network, thus could be promising for elucidating the disease therapies and guiding novel drug discovery in complex static network study.

### 4.2. Dynamic Network

**Figure 3:**
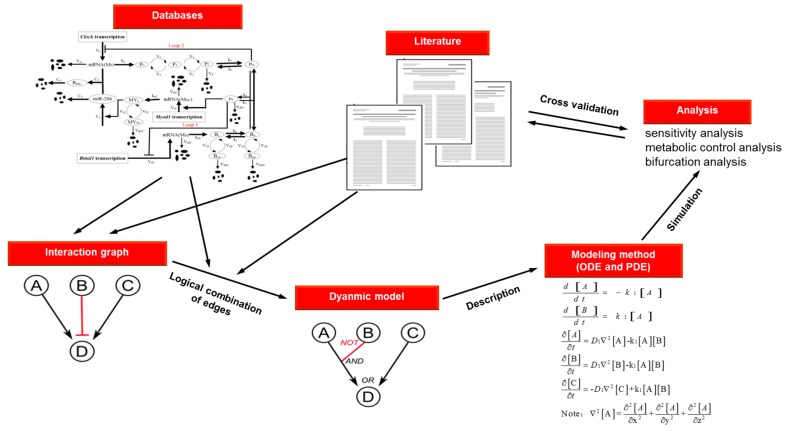
Modeling and simulation flow chart of dynamic network.

Unlike static networks, dynamic ones are networks whose structure may change in terms of time-series depending on various factors. The dynamic networks are more challenging compared with static network, since dynamic network obliges temporally, sometimes spatially resolves data or even more data. The descriptions of dynamic networks usually include edge directions, signs, conditionality and many dynamically changing quantitative measures. As a new source to promote the development of novel drugs, dynamic network simulation and analysis not only help us understand the dynamic behavior of key actors in space and time, but also provide better insights into the predicting drug targets and their role in human pathophysiology. The modeling and simulation flowchart of dynamic network is shown in Figure 3.

In general, dynamic network modeling is considered to be a series of chemical reactions, whose kinetics can be described by ordinary differential equation (ODE) and partial differential equation (PDE) to execute deterministic and stochastic simulations (Table 3). A system of ODEs is the most fundamental way to quantitatively simulate the dynamic response of each components in a network under different conditions [76], which can either be figured out exactly or by an approximate analytical method. The network modeled with deterministic ODEs can easily investigate steady states and the varieties of dynamic behavior with the state of system. Despite compartmental ODE modeling frequently used as a simplification for PDEs [77], sometimes detailed spatial localization is critical for cell signaling networks [78,79]. In this case, as reaction-diffusion equations in biochemical processes, PDEs can be used to explain diffusion and biochemical reactions of signaling markers in network. Under such situations, a stochastic model based on PDEs that allows for inherent fluctuations in dynamic network may give rise to qualitatively different behavior which differs significantly from those predicted by deterministic models. Different from the deterministic implementation, the stochastic simulation reactant equations were considered to interact as discrete entities. The explanation of random fluctuations which possibly affect reaction dynamics can be achieved [80]. The increased concern on the significance of signaling noise and the proliferation of single cell measurements proposed that stochastic models will be more accepted due to the potential availability and replicability of the variation of individual cell responses.

Given a dynamic network, one can characterize the system behaviors using various analysis approaches, such as sensitivity analysis, metabolic control analysis and bifurcation analysis. These methods are useful for exploring the potential system dynamics and quantitative insights into emergent system behaviors, such as robustness. Sensitivity analysis is used to quantify changes in system behaviors with respect to parametric perturbations [81]. In a complex system with a large number of parameters, different parameters may have various impacts on the system dynamics. Sensitivity analysis which requires the most precise measurement provides a way to select parameters with greatest influence on the production of system. [82]. Similar to sensitivity analysis, bifurcation analysis also focuses on a qualitative understanding of the system dynamics and performs by varying a parameter until a qualitative change in dynamics is observed. Bifurcation analysis is favorable in comprehending the transitions between dynamic behaviors due to the changes in model parameters. Metabolic control analysis develops the mathematical and theoretical framework to outline the quantitatively control method for a specific enzyme which functions on flux and the concentration of metabolites, consequently substituting the intuitive and qualitative limitation concept. It provides a conceptual framework for understanding the control of fluxes through metabolic pathways at the molecular level. It is a valuable post-genomic tool to handle systems of any complexity and does not need all system components to be known previously. By applying these approaches, it is possible to identify the important steps or key factors that should have significant alteration on flux or concentration in pathways, so as to understand the complex mechanism of disease and to predict target for developing novel drugs.

## 5. Conclusions

The drug discovery process is a time consuming and complex process requiring multi-disciplinary approaches to develop riskless and effective medicines. Despite the great synthetic diversity derived from the development of combinatorial chemistries and high-throughput screening methods, they have had notably small influence on the derivation of novel drugs and candidate compounds for primary optimization [83,84]. Post-marketing failures of blockbuster drugs remain recognized as extremely important elements in the pharmaceutical industry. Contemporary clinical knowledge and experiential databases are helpful in raising success rate by lessening the time wasted, money spent and diverse effect occurrence, which are the leading bottlenecks in drug development in contrast to ordinary approach integrating various technologies for screening from small molecule compounds. However, due to the complexity of the interactions between drugs and their targets, a quick search and understanding of therapeutic molecules based on the traditional method is a massive challenge. These call for systematic and critical reviews of methods and mindsets involved in drug discovery today, which must overcome problems above and become more integrated, fast, focused and predictive, where safety and efficacy issues are addressed alongside the developmental costs [85].

The present review focuses on the concepts of innovative drug discovery rather than on specific pharmaceutical techniques and knowledge. This review aims to outline accessible information on current methods and strategies in novel especially complementary and alternative drug discovery. Moreover, it will benefit individualized therapy, which provides the chance to improve therapeutic efficacy targeting the genomic aberrations in disease states as well as reducing the undesirable toxicity due to the alteration of drug metabolism based on the patients’ genotype. Such an advance contributes to diagnostic tests recording benefits of individualized medicine on certain patients. Our strategy in this review will not only lead to saving of expenditure and time, associated with increased success rate in small molecule drug discovery and development, but also be considered to minimize the risk of post-marketing withdrawals and go a long way in safeguarding the interests of both pharmaceutical industry and ordinary civilians.

## Figures and Tables

**Table 1:** The methods of drug–target interactions prediction.

Method	Description	Advantages	Disadvantages
Ligand-based	Based on the similarity of known ligands	Applicable when the structure of the receptor site is unknown	Not applicable when no ligands for a given protein exist
Structure-based	Based on binding of ligands to active sites of the target protein	Rich information on various target proteins	Not applicable to proteins whose 3D structures are unknown
Phenotype-based	Based on the desired biological phenotypic information	Applicable to the genome-scale computation	Possibly ignore valuable computation from other types of data sources

**Table 2:** Important topological characteristics in static network.

Network Characteristics	Definition	Biological Entities and Functions
Node	Basic component interacting (pair-wise) with other node(s)	Small-molecular (metabolic network)
		Genes (genetic regulatory network)
		Proteins (protein-protein network)
Edge	A relationship between the nodes	Connection may be physical, regulatory, genetic interaction
		Metabolic network: enzyme-catalyzed reactions
		Genetic regulatory network: expression data
Degree	Number of links to other nodes	Associated with topological robustness of biological networks *i.e.*, small degree nodes are more “disposable” than hubs
Betweenness	Number of shortest paths that pass through each node	Important for finding non-hub crucial nodes or classifying hubs according to their positions in the network
Closeness	Number of link to the center	Only applicable to connected networks
Eigenvector	Influence of a node in a network	Assigning relative scores to all nodes in the network

**Table 3:** Summary of different methods used in dynamic network.

Method	Description	Reaction	Equation	Advantages	Disadvantages
ODEs	Series of reaction-rate equations solved using numerical methods	$A\overset{k_{1}}{\to }B$	$\begin{array}{l} \frac{d\left[A\right]}{dt}=-k_{1}\left[A\right]\\ \frac{d\left[B\right]}{dt}=k_{1}\left[A\right] \end{array}$	Well understood formalism	Limited to temporal modeling
				Deterministic	Assumed high concentrations and uniform mixing
	Produces graphs or tables of reagent production and consumption			Fast	Brittle
				Mathematically robust	
PDEs	Expresses spatial and temporal dependence through partial derivatives	$A+B\overset{k_{1}}{\to }C$	$\begin{array}{l} \frac{\partial \left[A\right]}{\partial t}=D_{1}\nabla^{2}\left[A\right]-k_{1}\left[A\right]\left[B\right]\\ \frac{\partial \left[B\right]}{\partial t}=D_{1}\nabla^{2}\left[B\right]-k_{1}\left[A\right]\left[B\right]\\ \frac{\partial \left[C\right]}{\partial t}=-D_{1}\nabla^{2}\left[C\right]+k_{1}\left[A\right]\left[B\right]\\ \text{Note}:\nabla^{2}\left[A\right]=\frac{\partial^{2}\left[A\right]}{\partial x^{2}}+\frac{\partial^{2}\left[A\right]}{\partial y^{2}}+\frac{\partial^{2}\left[A\right]}{\partial z^{2}} \end{array}$	Well understood formalism	Complicated
				Possible to be fast	Difficult to implement or generalize
	Bases on numerical methods	With diffusion of molecules at rate D_1_		Mathematically robust	Unable to model state of discontinuous transitions
	Produces numeric output of concentrations and *x, y, z* coordinates			Enables modeling of time- and space-dependent process	Brittle
